# Supplementary material for: Transductomics: sequencing-based detection and analysis of transduced DNA in pure cultures and microbial communities
Source: Microbiome. 2020 Nov 15;8:158. doi: 10.1186/s40168-020-00935-5 (PMC7667829; doi:10.1186/s40168-020-00935-5)

Figure S2: Detection of generalized transduction and GTA-like read coverage patterns depending on contig length. Different contig length were simulated using the P22, P1 and PBSX transduction datasets.

a) Generalized transduction by phage P22

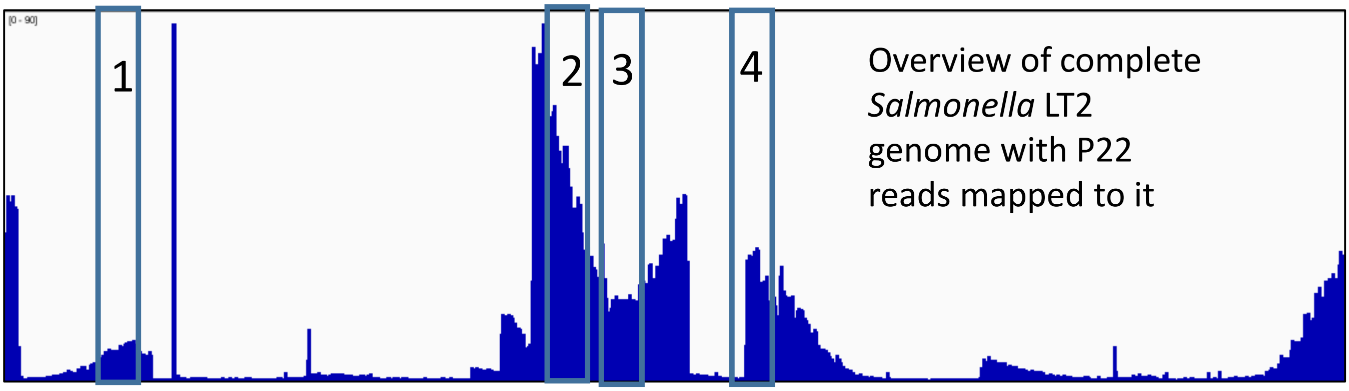

Enlargements of the four 200 kbp sections indicated above showing what the read coverage pattern would look like on a 200 kbp contig for specific regions within the generalized transduction pattern.

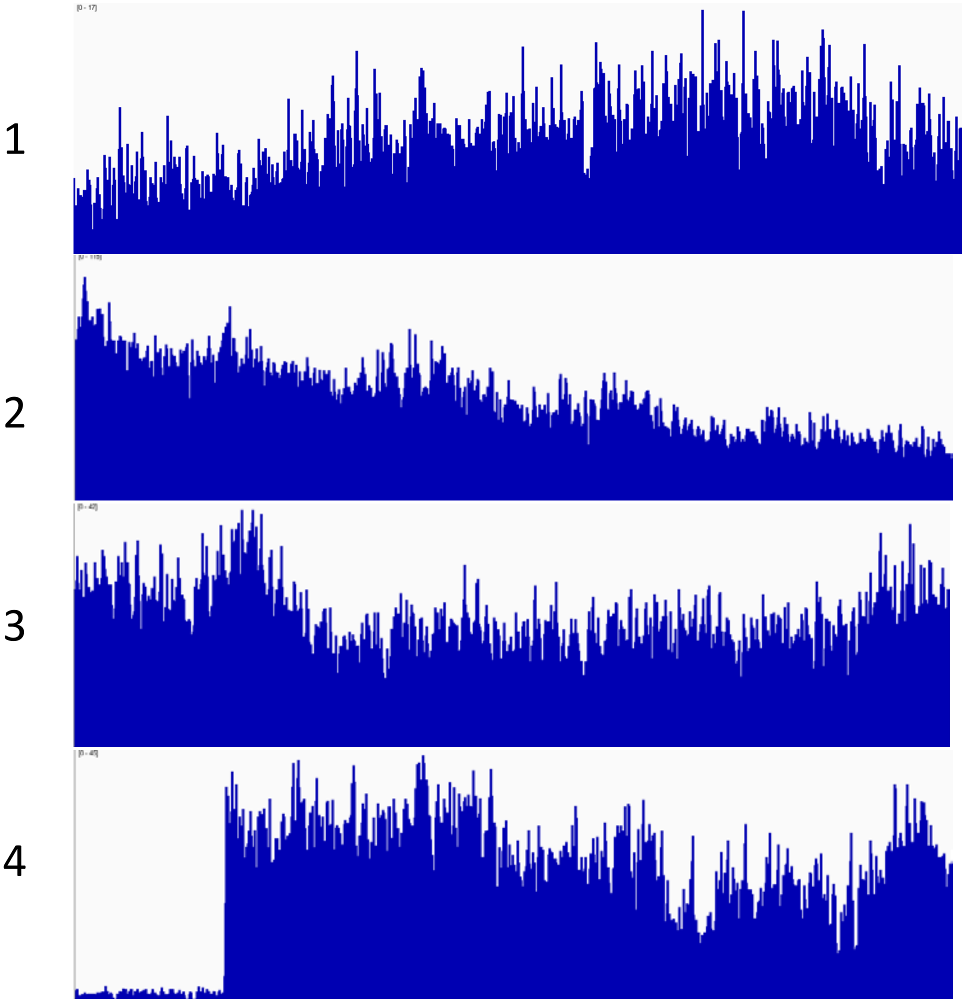

b) Generalized transduction by phage P1

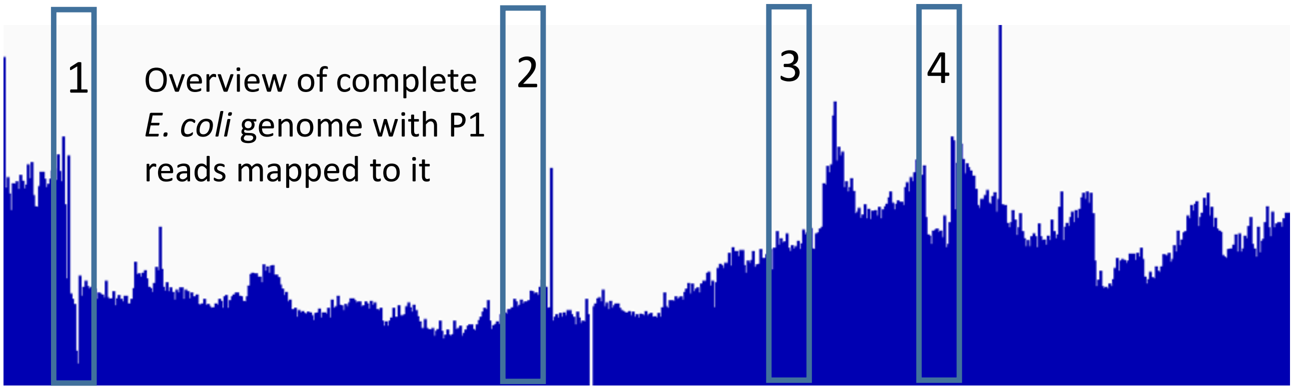

Enlargements of the four 200 kbp sections indicated above showing what the read coverage pattern would look like on a 200 kbp contig for specific regions within the generalized transduction pattern.

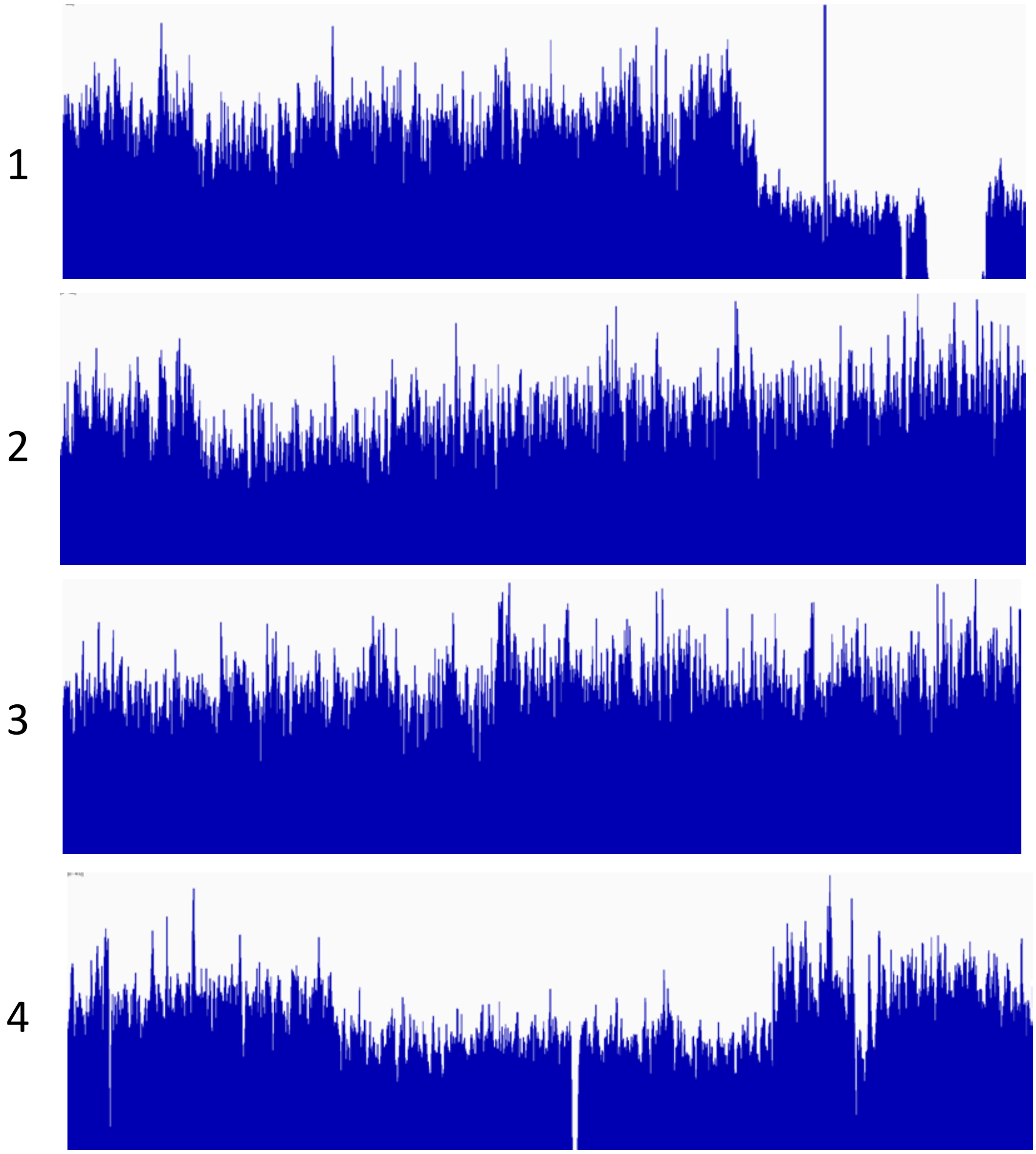

c) GTA-like DNA transport by defective prophage PBSX

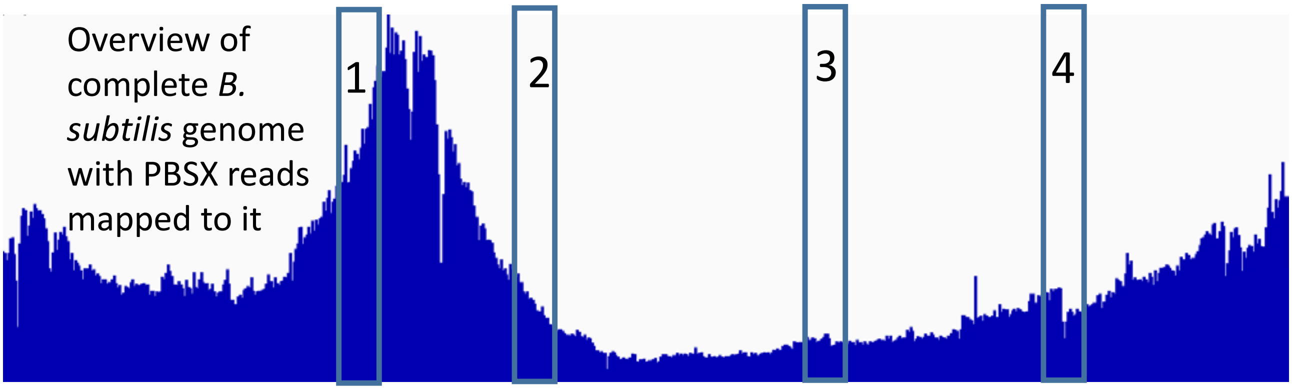

Enlargements of the four 200 kbp sections indicated above showing what the read coverage pattern would look like on a 200 kbp contig for specific regions within the generalized transduction pattern.

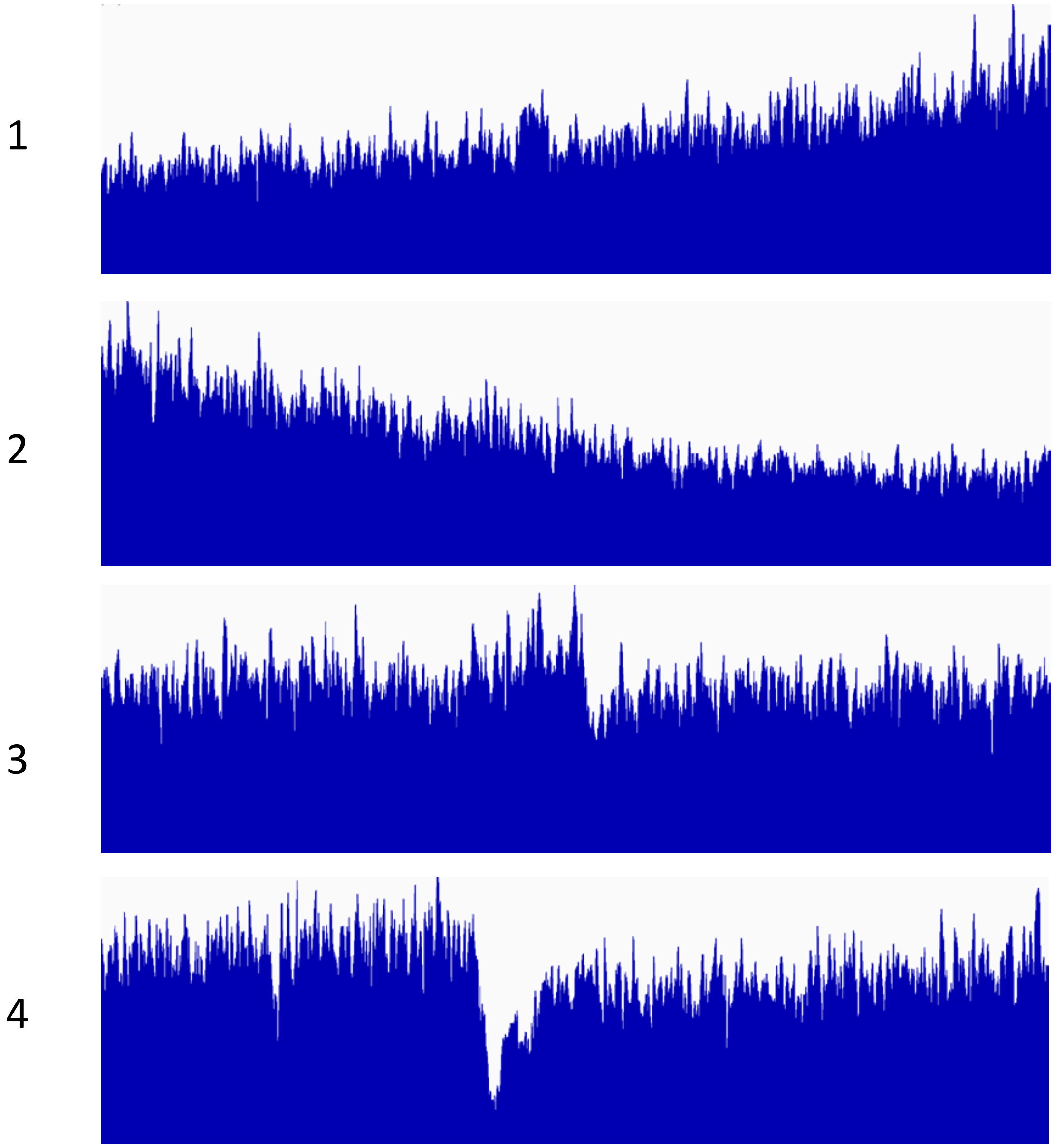

Supplement: Supplementary file 3 — Additional file 2: Figure S2. [file 40168_2020_935_MOESM2_ESM.pdf]
